# Trichophyton rubrum Azole Resistance Mediated by a New ABC Transporter, TruMDR3

**DOI:** 10.1128/AAC.00863-19

**Published:** 2019-10-22

**Authors:** Michel Monod, Marc Feuermann, Karine Salamin, Marina Fratti, Maya Makino, Mohamed Mahdi Alshahni, Koichi Makimura, Tsuyoshi Yamada

**Affiliations:** a Department of Dermatology, Centre Hospitalier Universitaire Vaudois, Lausanne, Switzerland; b Swiss-Prot Group, SIB Swiss Institute of Bioinformatics, Geneva, Switzerland; c Graduate School of Health Care Science, Bunkyo Gakuin University, Tokyo, Japan; d Graduate School of Medicine, Teikyo University, Tokyo, Japan; e Teikyo University Institute of Medical Mycology, Tokyo, Japan; f Asia International Institute of Infectious Disease Control, Teikyo University, Tokyo, Japan

**Keywords:** *Trichophyton rubrum*, dermatophytes, itraconazole, voriconazole, antifungal resistance, MFS transporters, ABC transporters

## Abstract

The mechanisms of terbinafine resistance in a set of clinical isolates of Trichophyton rubrum have been studied recently. Of these isolates, TIMM20092 also showed reduced sensitivity to azoles. The azole resistance of TIMM20092 could be inhibited by milbemycin oxime, prompting us to examine the potential of T. rubrum to develop resistance through multidrug efflux transporters. The introduction of a T. rubrum cDNA library into Saccharomyces cerevisiae allowed the isolation of one transporter of the major facilitator superfamily (MFS) conferring resistance to azoles (TruMFS1). To identify more azole efflux pumps among 39 ABC and 170 MFS transporters present within the T. rubrum genome, we performed a BLASTp analysis of Aspergillus fumigatus, Candida albicans, and Candida glabrata on transporters that were previously shown to confer azole resistance. The identified candidates were further tested by heterologous gene expression in S. cerevisiae. Four ABC transporters (TruMDR1, TruMDR2, TruMDR3, and TruMDR5) and a second MFS transporter (TruMFS2) proved to be able to operate as azole efflux pumps. Milbemycin oxime inhibited only TruMDR3. Expression analysis showed that both *TruMDR3* and *TruMDR2* were significantly upregulated in TIMM20092. TruMDR3 transports voriconazole (VRC) and itraconazole (ITC), while TruMDR2 transports only ITC. Disruption of *TruMDR3* in TIMM20092 abolished its resistance to VRC and reduced its resistance to ITC. Our study highlights TruMDR3, a newly identified transporter of the ABC family in T. rubrum, which can confer azole resistance if overexpressed. Finally, inhibition of TruMDR3 by milbemycin suggests that milbemycin analogs could be interesting compounds to treat dermatophyte infections in cases of azole resistance.

## Abstract (precis)

The mechanisms of terbinafine resistance in a set of clinical isolates of Trichophyton rubrum have been studied recently. Of these isolates, TIMM20092 also showed reduced sensitivity to azoles. The azole resistance of TIMM20092 could be inhibited by milbemycin oxime, prompting us to examine the potential of T. rubrum to develop resistance through multidrug efflux transporters.

## INTRODUCTION

Tinea pedis and tinea unguium are mainly caused by two anthropophilic dermatophyte species, Trichophyton rubrum and Trichophyton interdigitale, with prevalences in Europe of 80% and 20%, respectively (1). Allylamines and azoles, targeting the ergosterol biosynthetic pathway, are the main oral and topical pharmacological options used to treat dermatophytosis. Allylamines, such as terbinafine, are inhibitors of the squalene epoxidase (SQLE), which is involved in the early steps of ergosterol biosynthesis (2). Its inhibition results in the accumulation of squalene, which is toxic to fungi (3, 4). Azoles, such as itraconazole (ITC) and voriconazole (VRC), act downstream of the SQLE reaction in the membrane during ergosterol synthesis. These antifungal drugs inhibit lanosterol 14-α-demethylase, resulting in the accumulation of sterol precursors (5). Terbinafine and azole compounds have been extensively used to treat dermatophyte infections.

Dermatophytes with reduced sensitivity to antifungal drugs have emerged in several countries (Switzerland, Denmark, and India) (6–10). Terbinafine resistance was imputed to single point mutations within the *SQLE* gene in 17 T. rubrum clinical isolates from Switzerland, leading to amino acid substitutions at one of four amino acid positions (Leu^393^, Phe^397^, Phe^415^, and His^440^) within the SQLE enzyme. T. rubrum isolates resistant to terbinafine had comparable ITC and VRC MICs, except for one isolate (TIMM20092) which showed reduced susceptibility to azole compounds. This strain was isolated from a patient with tinea pedis insensitive to standard systemic and topical terbinafine and ITC treatments. Of note, resistance of the growing fungus to azoles could be reversed by a subinhibitory concentration of milbemycin oxime, an inhibitor of ABC transporters. These findings led to the hypothesis that the overexpression of genes that encode transporters could be involved in azole resistance.

While several Aspergillus and Candida azole transporters belonging to the ATP-binding cassette (ABC) transporters or the major facilitator superfamily (MFS) transporters have been well characterized, much less is known about the involvement of transporters in the azole resistance in dermatophytes. Gene expression data have shown that azoles and other drugs induce the transcription of some ABC transporters in dermatophytes, including MDR1/PDR1, MDR2, and MDR4 (11–13). However, the direct involvement of these transporters in azole efflux has yet to be shown. The isolation of a strain resistant to azoles, in addition to terbinafine, from a patient insensitive to standard treatment incited us to examine the potential of T. rubrum to develop azole resistance through multidrug efflux transporters.

## RESULTS

### Reduced sensitivity of strain TIMM20092 to azoles.

The MICs of VRC and ITC of TIMM20092 were measured at 0.063 μg/ml and 0.5 μg/ml, respectively, by the broth microdilution method. In the presence of 1.5 μg/ml milbemycin oxime, known to be an inhibitor of ABC transporters (14–16), the MIC of VRC decreased 75% to 0.015 μg/ml, and that of ITC decreased by 50% to 0.25 μg/ml. The values of 0.015 μg/ml (VRC) and 0.25 μg/ml (ITC) were close to those obtained for 10 clinical isolates with or without the presence of milbemycin oxime and are in line with values reported in the literature for T. rubrum (10, 17, 18). One of these clinical isolates, CHUV1845, was selected for comparison with TIMM20092 in further experiments.

**FIG 1:**
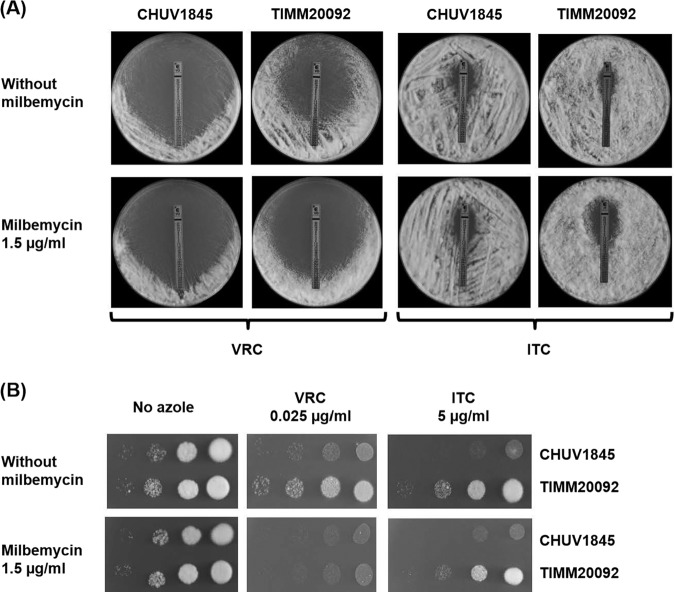
Susceptibility and resistance of T. rubrum strains, CHUV1845 and TIMM20092, to voriconazole (VRC) and itraconazole (ITC) with and without 1.5 μg of milbemycin oxime. (A) Etest strips laced with an azole derivative were placed on fungal lawns (10^6^ CFU) prepared on SDA plates. (B) Serial dilution drug susceptibility assays to VRC and ITC. T. rubrum spores were spotted at different dilutions on SDA plates, as described in Materials and Methods. The plates were incubated at 30°C for 7 days.

A reduced sensitivity of TIMM20092 to ITC, similar to VRC, was clearly demonstrated by Etests and by spot tests on Sabouraud dextrose agar (SDA) containing the antifungal drugs (Fig. 1). The sensitivity of TIMM20092 to VRC was increased by adding 1.5 μg/ml milbemycin oxime to the agar medium, while resistance to ITC was only slightly affected. We found that at this concentration, milbemycin oxime did not affect fungal growth.

We sequenced the *cyp51A* gene encoding the azole target in TIMM20092 and CHUV1845. The sequence of *cyp51A* was identical to that of the sequences from the genomes of strains ATCC MYA-4607/CBS 118892 and CBS 288.86 submitted to UniProtKB (accession identifiers [IDs] F2SHH3 and A0A022VVX6, respectively). These results indicated that the resistance of TIMM20092 could not be attributed to a missense mutation of the drug target.

### Identification of a new MFS antifungal transporter using a pool of plasmids from a previously constructed T. rubrum cDNA library.

**FIG 2:**
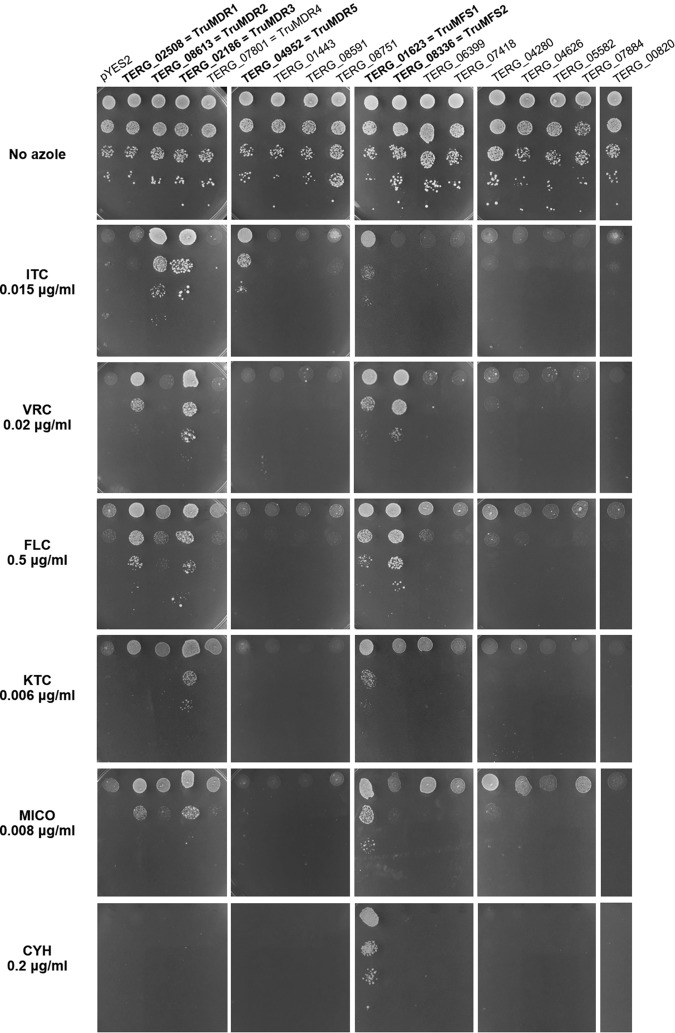
Susceptibility and resistance to itraconazole (ITC), voriconazole (VRC), fluconazole (FLC), ketoconazole (KTC), miconazole (MICO), and cycloheximide (CYH) of S. cerevisiae transformed with different plasmids encoding MFS and ABC transporters. The efflux pumps conferring drug resistance are highlighted in bold. Yeasts were spotted at different dilutions on MMG plates, as described in Materials and Methods. As a control, S. cerevisiae was spotted on plates without antifungal drugs. The plates were incubated at 30°C for 7 days. S. cerevisiae transformed with plasmid pYES2 was used as a control of susceptibility (top row).

To explore the molecular basis of antifungal drug resistance in dermatophytes, we first transformed Saccharomyces cerevisiae Y02409 with a pool of plasmids from a previously constructed cDNA library, where T. rubrum cDNA sequences were cloned into the pYES2-DEST52 vector downstream of the *GAL1* promoter for inducible, heterologous gene expression (19) (refer to Materials and Methods). Transformant cells were seeded on minimal medium with galactose (MMG) plates, which incorporated ITC and VRC at concentrations of 0.01 and 0.02 μg/ml, respectively. These concentrations were equivalent to about 5 times the MIC of S. cerevisiae Y02409 for both antifungal compounds. Five ITC-resistant and 12 VRC-resistant S. cerevisiae clones were obtained. The plasmid DNA of these clones was then isolated as previously described (20) and used for the transformation of Escherichia coli XL1-Blue. Sequence analysis revealed that all ampicillin-resistant E. coli clones obtained from each selected S. cerevisiae transformant contained a 2.1- to 2.3-kb cDNA insert with an identical 1.7-kb open reading frame (ORF). A BLAST search revealed that this ORF corresponded to TERG_01623, which encodes an MFS transporter. We designated this transporter TruMFS1. Unfortunately, no other transcripts containing a resistance gene were identified using this approach. TruMFS1 was able to transport all azoles tested, including miconazole (MICO), fluconazole (FLC), and ketoconazole (KTC). In addition, TruMFS1 was also able to transport cycloheximide (CYH) (Fig. 2).

### Identification of an additional T. rubrum MFS transporter involved in azole resistance.

A search in the T. rubrum proteome for proteins with a domain signature for MFS transporters led to the identification of 170 potential proteins in the UniProtKB database (https://www.uniprot.org/) (see Table S1 in the supplemental material). To restrict this list to transporters potentially involved in azole resistance, we took advantage of studies performed with the closely related pathogenic fungus Aspergillus fumigatus, as well as with the well-studied pathogenic yeasts Candida albicans and Candida glabrata. AFUA_1G13800/mdrA was the only A. fumigatus MFS transporter for which involvement in azole resistance has been demonstrated (21), while additional MFS transporter genes of A. fumigatus were reported to be induced either by VRC (*mfsB*) (22) or by ITC (*mdr3*) (23). The BLAST analysis led to the identification of three transporters, TERG_08336, TERG_07418, and TERG_06399, whose products are the closest homologs of A. fumigatus *mdrA*, *mfsB*, and *mdr3*, respectively. The BLAST analysis also showed that the closest homolog of TruMFS1 that we identified earlier was aflT, a transporter whose gene is part of the aflatoxin cluster in the *Aspergillus* species (24). Seven MFS transporters from both C. albicans and C. glabrata led to the identification of five additional T. rubrum MFS candidates (TERG_00820, TERG_04280, TERG_04626, TERG_05582, and TERG_07884) (Table 1). There was no overlap between the sets of transporter genes identified from *Aspergillus* and *Candida*.

**TABLE 1:** Identification of potential T. rubrum MFS transporters involved in azole resistance

Locus name by organism	Transporter name(s)/synonyms	Resistance(s)or substrate information^*a*^	PMID (reference)	T. rubrum homolog(s)^*c*^	Protein sequence identity, length of identical parts of proteins (aa)^*b*^
A. fumigatus					
AFUA_1G13800	mdrA	VRC and FLC	26933209 (21)	**TERG_08336 (TruMFS2)**	64.4, 595
AFUA_1G15490	mfsB	VRC induced	16622700 (22)	TERG_07418	65.1, 578
AFUA_4G10000	mdr3	ITC induced	15563516 (23)	TERG_06399	77.8, 803
AFUA_1G12620	aflT	Not aflatoxin	17620135 (24)	**TERG_01623 (TruMFS1)**	53.2, 494
C. albicans					
CAALFM_CR04210CA	QDR1	Not azoles	24621232 (62)	TERG_04280	28.1, 509
CAALFM_C305570WA	QDR2	Not azoles	24621232 (62)	TERG_04280	29.2, 504
CAALFM_C601290CA	QDR3	Not azoles	24621232 (62)	TERG_00820	45.8, 697
CAALFM_C701520WA	FLU1/TPO1	Mycophenolic acid, FLC	11065353 (63)	TERG_04626	49.5, 610
CAALFM_C603170CA	MDR1/BMR1/BEN1	Benomyl, CYH, methotrexate, FLC	8031026 (64), 9210670 (65), 15273122 (66), 10844673 (67), 11568478 (68)	TERG_05582	35.2, 564
CAALFM_C604610CA	NAG3/MDR97/TMP1	CYH	12076781 (69)	TERG_07884	56.4, 561
CAALFM_C604620CA	NAG4	CYH	12076781 (69)	TERG_07884	57.1, 581
C. glabrata					
CAGL0J09944g	AQR1	Acetic acid, flucytosine, CLT	23805133 (70)	TERG_04280	30.6, 492
CAGL0G08624g	QDR2	Quinidine, MICO, TIC, CLT, KTC	23629708 (71)	TERG_04280	30.1, 583
CAGL0G03927g	TPO1_1	CLT	26512119 (72)	TERG_04626	42.7, 518
CAGL0E03674g	TPO1_2	CLT	26512119 (72)	TERG_04626	48.3, 478
CAGL0H06017g	FLR1	5-Flucytosine	28066366 (73)	TERG_05582	35.5, 512
CAGL0H06039g	FLR2	5-Flucytosine	28066366 (73)	TERG_05582	35.7, 566
CAGL0I10384g	TPO3	CLT, MICO, KTC, FLC	24576949 (74)	TERG_07884	41.2, 450

a Compounds that are found to be substrates or to induce gene expression and those that are not substrates are indicated. ITC, itraconazole; VRC, voriconazole; FLC, fluconazole; MICO, miconazole; TIC, tioconazole; CLT, clotrimazole; KTC, ketoconazole; CYH, cycloheximide.

b aa, amino acids.

c The efflux pumps conferring drug resistance are named between parentheses and highlighted in bold.

Full-length amino acid sequences of the identified transporters in T. rubrum were confirmed or deduced by alignment against a set of high-quality transporter amino acid sequences, namely, protein sequences of the model organisms S. cerevisiae, Aspergillus nidulans, A. fumigatus, C. albicans, and Trichophyton benhamiae (formerly Arthroderma benhamiae), which were reviewed by UniProtKB/Swiss-Prot. Subsequently, we cloned DNA sequences encoding the eight MFS transporters into pYES2 and transformed S. cerevisiae with the generated plasmids. Yeast transformants were then tested for resistance to ITC, VRC, FLC, KTC, and MICO, as described in Materials and Methods, to examine the capability of each transporter to operate as an efflux pump. Only the product of TERG_08336, designated TruMFS2, was able to provide VRC and FLC resistance but not ITC, KTC, or MICO resistance (Fig. 2). TruMFS2 was close to the A. fumigatus transporter mdrA (21). Surprisingly, none of the T. rubrum homologs of *Candida* MFS transporters used for the BLAST analysis appeared to be involved in resistance to any of the azoles tested (Fig. 2).

### Identification of four T. rubrum ABC transporters involved in azole resistance.

A search in the proteome of T. rubrum in UniProtKB for proteins with a domain signature for ABC transporters led to the identification of 39 potential candidates (Table S2). Six of them were not actually transporters but derived from the ABC family since they have lost their transmembrane domains. Based on the literature, we screened 10 A. fumigatus, 4 C. albicans, and 3 C. glabrata transporters directly involved in azole resistance or induced by azoles (Table 2). The BLAST analysis identified 10 T. rubrum ABC transporters putatively involved in azole resistance. The only ABC transporter that did not have a homolog with >35% identity in T. rubrum was A. fumigatus atrF (AFUA_6G04360). In contrast, the products of TERG_02508 and TERG_02186, identified in T. rubrum, had homologs with >35% identity over a sequence of 1,000 amino acids in the three pathogenic fungi used for identification (Table 2). A. fumigatus mdr1/abcA (AFUA_5G06070) alone led to the identification of six different potential transporters of T. rubrum.

**TABLE 2:** Identification of potential T. rubrum ABC transporters involved in azole resistance

Locus name by organism	Transporter name(s)/synonyms	Resistance(s)or substrate information^*a*^	PMID (reference)	T. rubrum homologs^*c*^	Protein sequence identity, length of identical parts of proteins (aa)^*b*^
A. fumigatus					
AFUA_3G07300	atrI	ITC, VRC	26933209 (21)	**TERG_02508 (TruMDR1)**	58.6, 1,445
AFUA_1G14330	abcC/abcG1/cdr1B/atrE	ITC, VRC, POS	16622700 (22), 23580559 (36), 22509997 (75)	**TERG_02186 (TruMDR3)**, **TERG_02508 (TruMDR1)**	61.7, 1,084; 50.8, 1,404
AFUA_2G15130	abcA_2	FLC	24123268 (76), 23796749 (77)	**TERG_02186 (TruMDR3)**, **TERG_02508 (TruMDR1)**	71.5, 1,085; 50.8, 1,404
AFUA_5G06070	mdr1/abcA	VRC induced	16622700 (22)	**TERG_08613 (TruMDR2)**, TERG_08693, TERG_00402, TERG_08751, **TERG_04952 (TruMDR5)**, TERG_08591	68.5, 1,318; 50.1, 1,032; 49.1, 434; 41.7, 1,349; 40.7, 995; 38.7, 1,349
AFUA_6G03470	fmpD	Fumipyrrole	25582336 (78)	**TERG_08613 (TruMDR2)**	44.1, 1,279
AFUA_1G17440	abcA	Azoles (predicted)	12172968 (79)	**TERG_02186 (TruMDR3)**	57.0, 1,074
AFUA_1G10390	abcB	VRC induced	16622700 (22)	TERG_01443	59.3, 933
AFUA_1G12690	mdr4	VRC	21321135 (25)	TERG_07801 (TruMDR4)	60.0, 1,317
AFUA_7G00480	abcE	VRC induced	16622700 (22)	TERG_08751, **TERG_04952 (TruMDR5)**	53.8, 1,269; 39.2, 936
AFUA_6G04360	atrF	VRC	26933209 (21)	—^*d*^	
C. albicans					
CAALFM_C603840CA	SNQ2	Benomyl induced	15273122 (66)	**TERG_02508 (TruMDR1)**	42.8, 1,397
CAALFM_C305220WA	CDR1	FLC	8585712 (34)	**TERG_02186 (TruMDR3)**	45.3, 1,409
CAALFM_C304890WA	CDR2	Various azoles	9043118 (35)	**TERG_02186 (TruMDR3)**	44.7, 1,372
CAALFM_C108070WA	CDR4	Not FLC, CAS induced	9767132 (80), 15917516 (81)	**TERG_02186 (TruMDR3)**	45.3, 1,463
C. glabrata					
CAGL0I04862g	SNQ2	FLC	18312269 (82)	**TERG_02508 (TruMDR1)**	40.6, 1,495
CAGL0M01760g	CDR1	FLC	11257032 (36)	**TERG_02186 (TruMDR3)**	40,3, 1,506
CAGL0M01760g	PDH1/CDR2	FLC	11257032 (36)	**TERG_02186 (TruMDR3)**	45.3, 1,408

a Compounds that are found to be substrates or to induce gene expression and those that are not substrates are indicated. ITC, itraconazole; VRC, voriconazole; FLC, fluconazole; POS, posaconazole; CAS, caspofungin. *A. fumigatus* abcA has been predicted to transport azoles by similarity but targeted disruption experiments did not confirm it.

b aa, amino acids.

c The efflux pumps conferring drug resistance are named between parentheses and highlighted in bold.

d No homolog of *A. fumigatus* atrF has been found in *T. rubrum*.

As for MFS transporters, we checked the full-length amino acid sequences by comparing the T. rubrum protein sequences with those of the model organisms S. cerevisiae, A. nidulans, A. fumigatus, C. albicans and *T. benhamiae*. In particular, we made corrections in the intron-exon structure of the gene encoding TruMDR1 based on the alignment of the polypeptide sequence of this transporter with those of the closest transporters in the aforementioned organisms. The accession number of the updated sequence encoding TruMDR1 is GenBank accession no. MK787254. For two transporters encoded by TERG_08693 and TERG_00402, the full-length protein sequence could not be recovered, suggesting that they probably corresponded to products of pseudogenes, and they were not considered further in this study.

The eight remaining ABC transporters were tested in S. cerevisiae for their ability to provide resistance to azoles (Table 3). Four candidates, encoded by TERG_02508, TERG_08613, TERG_02186, and TERG_04952, were able to render the yeasts resistant to one or several azoles. TERG_02508 and TERG_08613 correspond to TruMDR1 and TruMDR2, respectively, which were previously reported (10, 11). However, for the first time, our study shows their direct involvement in the transport of azoles. The expression of *TruMDR1* cDNA led to VRC, FLC, and MICO resistance, whereas the expression of *TruMDR2* cDNA led to only ITC resistance (Fig. 2). The product of TERG_02186 was the only ABC transporter gene that rendered the yeasts resistant to all azoles tested and was designated TruMDR3 (Fig. 2). The product of TERG_04952, designated TruMDR5, gave clear resistance to ITC but not to other azoles (Fig. 2). The expression of TERG_07801, coding for TruMDR4, closely related to the A. fumigatus VRC-resistant transporter, mdr4 (25), surprisingly yielded no resistance, as was the case for TERG_01443, TERG_08591, and TERG_08751 (Fig. 2).

**TABLE 3:** Characterization of potential T. rubrum transporters involved in azole resistance

Locus name by type	Transporter^*a*^ name	Resistance for azole^*b*^:						Comments^*c*^
		VRC	ITC	KTC	FLC	MIC	CYH	
ABC transporters								
TERG_02508	TruMDR1	+	−	−	+	+	−	Close to A. fumigatus, C. albicans, and C. glabrata azole transporters
TERG_08613	TruMDR2	−	+	−	−	−	−	Close to A. fumigatus mdr1
TERG_02186	TruMDR3	+	+	+	+	+	−	Close to A. fumigatus, C. albicans, and C. glabrata azole transporters
TERG_07801	TruMDR4	−	−	−	−	−	−	Close to A. fumigatus mdr4
TERG_04952	TruMDR5	−	+	−	−	−	−	Close to A. fumigatus abcE
TERG_01443	−	−	−	−	−	−	−	Close to A. fumigatus abcB
TERG_08591	−	−	−	−	−	−	−	Close to A. fumigatus mdr1
TERG_08751	−	−	−	−	−	−	−	Close to A. fumigatus mdr1 and abcE
MFS transporters								
TERG_01623	TruMFS1	+	+	+	+	+	+	Close to A. fumigatus aflatoxin transporter aflT
TERG_08336	TruMFS2	+	−	−	+	−	−	Close to A. fumigatus mdrA
TERG_07418	−	−	−	−	−	−	−	Close to A. fumigatus mfsB
TERG_06399	−	−	−	−	−	−	−	Close to A. fumigatus mdr3
TERG_04626	−	−	−	−	−	−	−	Close to C. albicans FLU1 and C. glabrata TPO1_1 and TPO1_2
TERG_07884	−	−	−	−	−	−	−	Close to C. albicans MDR1 and C. glabrata FLR1 and FLR2
TERG_05582	−	−	−	−	−	−	−	Low similarities with C. albicans NAG3 and NAG4 and C. glabrata TPO3
TERG_04280	−	−	−	−	−	−	−	Low similarities with C. albicans QDR1 and QDR2 and C. glabrata AQR1 and QDR2
TERG_00820	−	−	−	−	−	−	−	Low similarities with C. albicans QDR3

a Only transporters showing azole transporter activity have been named. The others are marked with a minus sign.

b +, Constructs showing resistance in S. cerevisiae; −, no resistance.

c Close homologs from A. fumigatus and/or *Candida* spp. are indicated.

### Milbemycin oxime inhibits TruMDR3.

**FIG 3:**
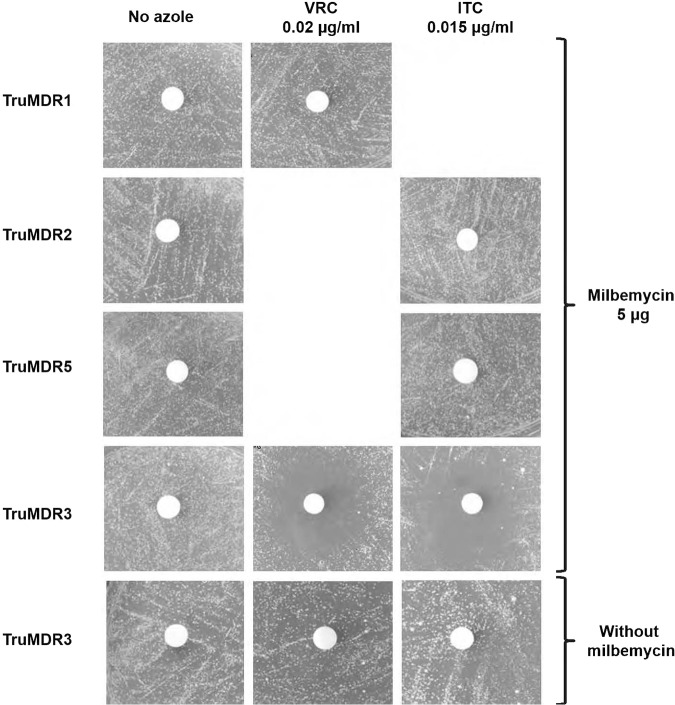
The effect of milbemycin oxime on TruMDR1, TruMDR2, TruMDR3, and TruMDR5. A total of 10^6^ cells of S. cerevisiae transformants were seeded to make a lawn on MMG plates with a sub-MIC of ITC (0.5 μg/ml) or VRC (0.64 μg/ml) incorporated into the medium. Nonimpregnated cellulose disks, 6 mm in diameter, were then placed at the surface of the medium and individually saturated with 5 μg of milbemycin oxime. The plates were incubated at 30°C, and growth inhibition was observed after 5 days. Growth inhibition was recorded for TruMDR3. No effect was observed for TruMDR1, TruMDR2, and TruMDR5, or for TruMFS1 and TruMFS2 (not shown). Milbemycin oxime has only been tested when the transporter induced resistance to VRC or ITC.

Using disk tests, we tested known inhibitors of ABC transporters, milbemycin oxime, ibuprofen, farnesol, haloperidol, promethazine hydrochloride, and curcumin (26–30), on the S. cerevisiae transformants, expressing individual transporters. S. cerevisiae cells were seeded to make a lawn on inducing MMG in the presence and absence of sub-MICs of VRC (0.02 μg/ml) or ITC (0.015 μg/ml). Cellulose disks were then placed on the surface of the medium and loaded with 5 μg of each inhibitor tested. Transformants expressing TruMDR3 were inhibited by milbemycin oxime (Fig. 3) but not by ibuprofen, farnesol, haloperidol, promethazine hydrochloride, or curcumin in the presence of either ITC or VRC (data not shown). Transformants expressing the genes encoding TruMDR1, TruMDR2, and TruMDR5 and the two MFS transporters TruMFS1 and TruMFS2 were not sensitive to milbemycin oxime and the other tested inhibitors. We concluded that milbemycin oxime was a specific inhibitor of TruMDR3.

### *TruMDR2* and *TruMDR3* are overexpressed in the azole-resistant strain TIMM20092.

**FIG 4:**
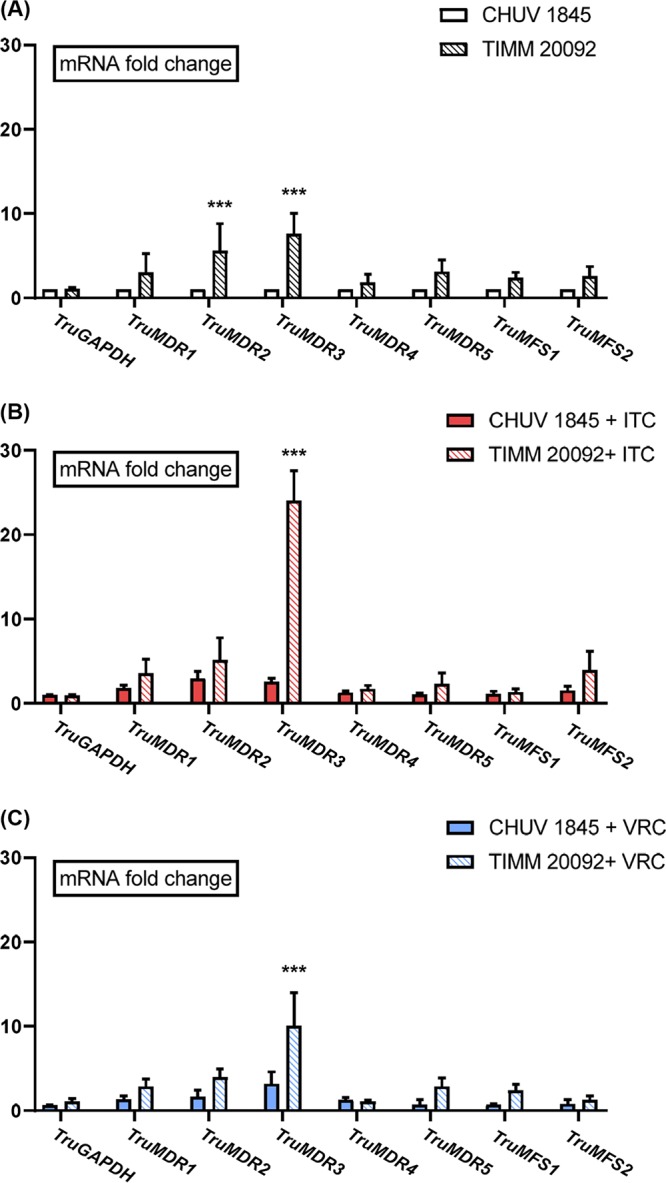
(A to C) Expression levels of genes encoding ABC and MFS transporters, as determined by qRT-PCR, in the absence of azole (A), upon exposure to itraconazole (ITC) (B), and upon exposure to voriconazole (VRC) (C). T. rubrum strains CHUV1845 and TIMM20092 were cultured for 7 days in SDB without an antifungal drug or SDB containing subinhibitory concentrations of ITC or VRC. The fold change represents the level of gene expression compared with that of T. rubrum CHUV1845 grown in SDB without antifungal drugs. The bars represent the standard deviation of the data obtained from three independent experiments. ***, *P* < 0.001.

We examined the expression of transporter genes in the azole-resistant strain TIMM20092 and in CHUV1845 (azole-sensitive strain) using quantitative real-time reverse transcription-PCR (qRT-PCR). The basal expression of *TruMDR2* and *TruMDR3* increased 5- and 8-fold, respectively, in TIMM20092, compared to that in CHUV1845 (Fig. 4A). The other transporter genes (*TruMDR1*, *TruMDR4*, *TruMDR5*, *TruMFS1*, and *TruMFS2*) were expressed 2- to 4-fold more in TIMM20092 than in CHUV1845.

ITC and VRC exposure increased significantly the transcription of *TruMDR3* in both TIMM20092 and CHUV1845 (Fig. 4B and C). A slight increase in the expression of *TruMDR1* and *TruMDR2* was also observed upon ITC exposure in CHUV1845 but not in TIMM20092. There was no significant difference in the expression of *TruMDR4*, *TruMDR5*, *TruMFS1*, and *TruMFS2* upon exposure to ITC or VRC in both strains.

### Disruption of *TruMDR3* in azole-resistant strain TIMM20092 leads to VRC sensitivity.

**FIG 5:**
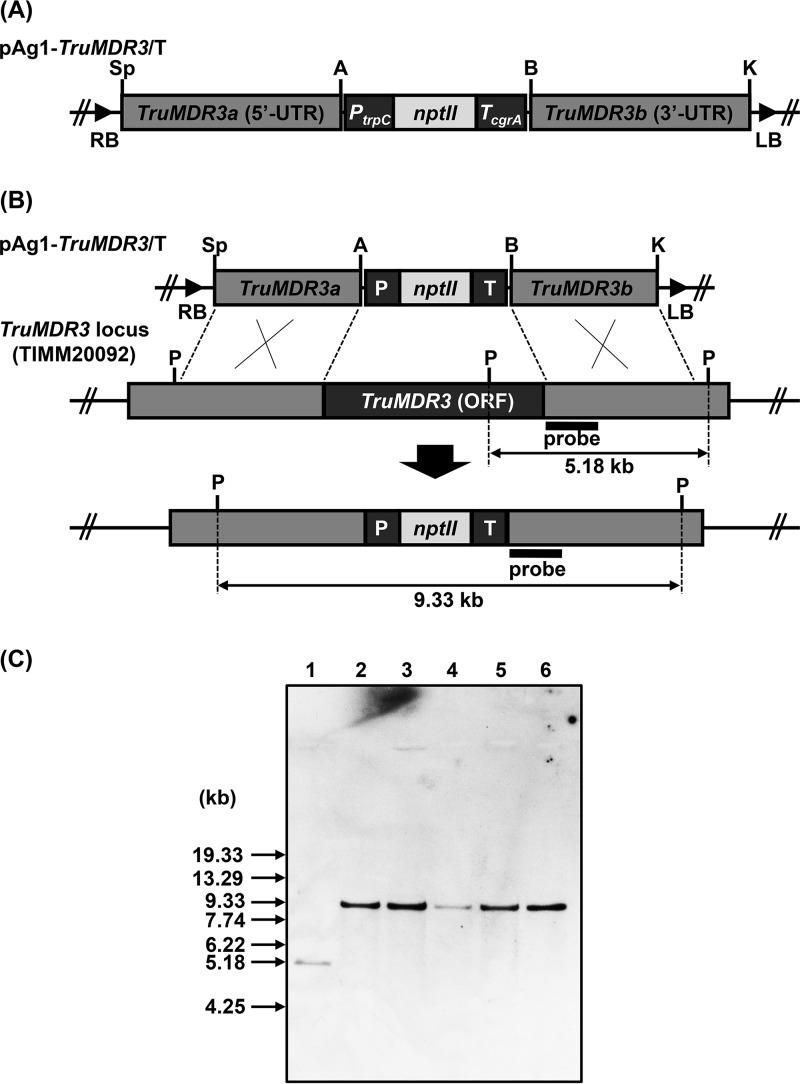
Disruption of the *MDR3* (*TruMDR3*) gene of T. rubrum TIMM20092 by gene replacement strategy. (A) Schematic representation of the binary *TruMDR3*-targeting vector pAg1-*TruMDR3*/T. The *nptII* cassette is composed of Aspergillus nidulans *trpC* promoter (*P_trpC_*), E. coli neomycin phosphotransferase gene (*nptII*), and the A. fumigatus *cgrA* terminator (*T_cgrA_*). LB and RB, left and right borders, respectively; A, ApaI; B, BamHI; K, KpnI; P, PstI; S, SpeI. (B) Schematic representation of the *TruMDR3* locus before and after homologous recombination. (C) Southern blotting. Lane 1, TIMM20092 (parent strain); lanes 2 to 6, ΔTruMDR3-11, -22, -33, -39, and -45, respectively. A 509-bp fragment of the *TruMDR3* locus was amplified by PCR with a pair of the primers P35 and P43 (Table S4) and used as a hybridization probe. DNA standard fragment sizes are shown on the left.

In order to examine the importance of *TruMDR3* in azole resistance of TIMM20092, we carried out the disruption of the *TruMDR3* locus by a gene replacement strategy (Fig. 5A and B). Since TIMM20092 is a wild-type clinical isolate, the targeting frequency of the *TruMDR3* locus by homologous recombination in this strain was low. Of 150 G418-resistant transformants obtained by the A. tumefaciens-mediated transformation, 5 transformants have been found to be the desired *TruMDR3* disruptants by PCR and Southern blotting (Fig. 5C).

**FIG 6:**
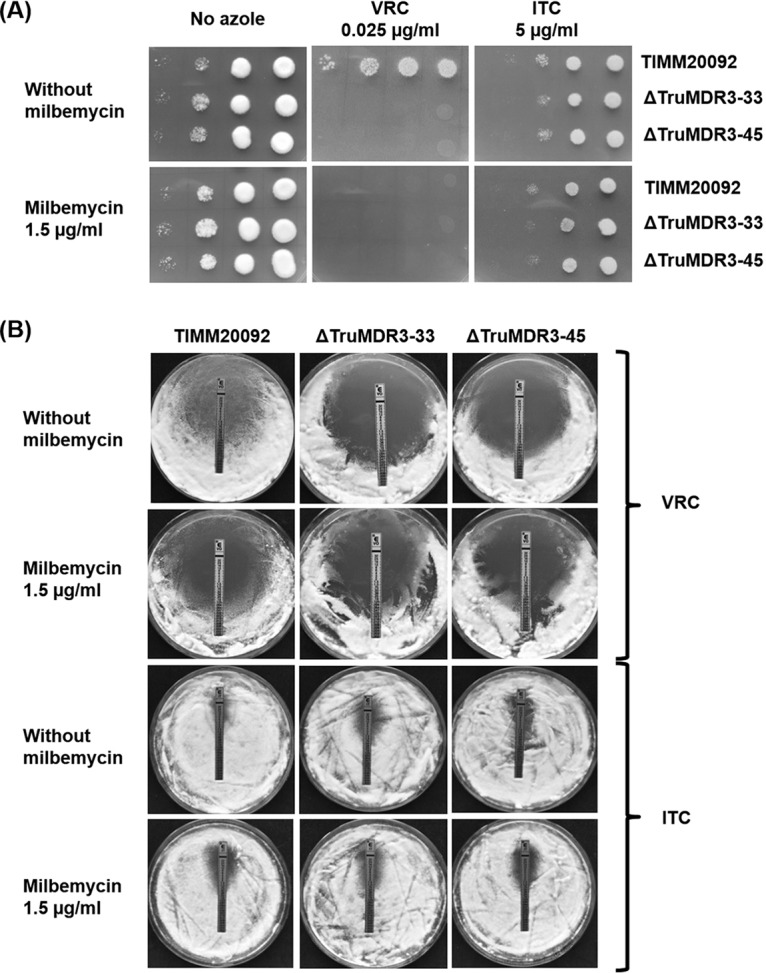
Susceptibility and resistance of T. rubrum strain TIMM20092, and two *TruMDR3* mutants, ΔTruMDR3-33 and ΔTruMDR3-45, to voriconazole (VRC) and itraconazole (ITC). (A) Serial dilution drug susceptibility assays to VRC and ITC. T. rubrum spores were spotted at different dilutions on SDA plates, as described in Materials and Methods. The plates were incubated at 30°C for 7 days. (B) Etest strips laced with VRC and ITC were placed on fungal lawns (10^6^ CFU) prepared on SDA plates.

Spot tests on SDA medium and Etests show that the deletion of *TruMDR3* in TIMM20092 mimics the addition of milbemycin and abolishes the resistance to VRC (Fig. 6A). This was confirmed by Etest (Fig. 6B). The deletion of *TruMDR3* affected the resistance to ITC to a lesser extent, suggesting that another transporter could also be involved in ITC resistance of TIMM20092 (Fig. 6A and B).

## DISCUSSION

For the first time, to our knowledge, we have described mechanisms of resistance to azole compounds in T. rubrum. Several mechanisms have been shown to be involved in nondermatophyte pathogenic fungi. These include the overexpression of the gene encoding lanosterol-14α-demethylase (*erg11* in *Candida*, *cyp51A* in *Aspergillus*), or its variant (23, 31–33), and the overexpression of several multidrug efflux transporters (31, 33–38). We found in T. rubrum four ABC transporters, TruMDR1, TruMDR2, TruMDR3, and TruMDR5, and two MFS transporters, TruMFS1 and TruMFS2, able to act as efflux pumps for at least one of the azole compounds tested. TruMDR3 and TruMFS1 were able to transport all five azoles tested, showing them to have a broad specificity toward azoles. In addition, TruMFS1 has also been shown to be active toward CYH, suggesting that its substrate specificity is not restricted to azoles but is broader and could be considered a pleiotropic drug transporter. In contrast, TruMDR1 and TruMFS2 have been found to be efflux pumps for VRC and FLC (Fig. 2), which have similar molecular structures. The genes coding for two transporters, TruMDR2 and TruMDR3, were found to be overexpressed in a clinical isolate which showed reduced sensitivity to azoles, TIMM20092. Unlike terbinafine resistance in dermatophytes, the reduction in sensitivity of TIMM20092 to azoles was not mediated by a missense mutation in the antifungal drug target.

### Antifungal susceptibility testing.

Spot tests, Etests, and microdilution assays were performed with adaptations from the Clinical and Laboratory Standards Institute (CLSI) guidelines (39) (use of SDA medium instead of RPMI 1640 and spectrophotometric measure of fungal growth) when working with T. rubrum, which was more experimentally demanding than other sporulating, fast-growing filamentous fungi (e.g., A. fumigatus) (40). The growth of T. rubrum isolates was faster, and mycelium was more abundant in Sabouraud dextrose broth (SDB) than in RPMI 1640 liquid or agar medium. Differences between MIC values in liquid and agar media were recorded for the same isolate. However, regardless of the method used, TIMM20092 was always more resistant than all other isolates for which the MIC appeared to be similar. The MIC of TIMM20092 always deviated from the standard MIC of the other strains.

### Comparison of T. rubrum azole transporters with closest A. fumigatus homologs.

ITC is extensively used in therapy for dermatophyte infections (41). Recently, VRC, routinely used in clinical practice to treat fungal infections of the central nervous system (CNS) (42), was shown to be effective in treating dermatophytosis (43). Therefore, we first tested ITC and VRC on T. rubrum in our research on azole resistance involving transporters. Our first attempt to isolate dermatophyte azole transporters, using a pool of plasmids from a previously established cDNA library of T. rubrum, only led to the identification of an MFS type transporter, TERG_01623, whose closest homolog in *Aspergillus* is aflT. The gene encoding aflT is located within the aflatoxin gene cluster in *Aspergillus* species (24). The expression of the *aflT* gene in Aspergillus parasiticus is not controlled by the aflatoxin pathway-specific activator aflR, nor by the coactivator aflJ, and aflT does not have a significant role in aflatoxin secretion (44). It has not been demonstrated whether aflT plays a role in azole efflux. No other homologs of previously known *Candida* or *Aspergillus* azole transporters were isolated in the screen. This comes as no surprise since the cDNA bank used in this study was not produced under stress conditions with azoles that could induce the expression of genes encoding efflux pumps. In addition, the large size of transporters such as ABC transporters may have prevented them from being present in the library. To select additional azole transporter candidates in T. rubrum, we searched for homologs of azole transporters characterized in the well-studied fungal pathogens A. fumigatus, C. albicans, and C. glabrata and found five additional azole transporters among 16 candidates selected by the BLAST search.

Differences in substrate specificity were observed between azole transporters in A. fumigatus and their closest homologs in T. rubrum (Table 3). A. fumigatus mdr1/abcA has six 40 to 60% identical homologs in T. rubrum, but only two of them (TruMDR2 and TruMDR5) showed azole transport activity (Table 2). TruMDR2 is closest to A. fumigatus mdr1, while TruMDR5 shared a lower identity with A. fumigatus mdr1 than did TERG_08693, TERG_00402, and TERG_08751 products, which did not show any ITC or VRC transport activity. A difference in substrate specificity was also observed between TruMDR4 and its A. fumigatus homolog, mdr4. TruMDR4 showed no activity toward any of the five azoles tested (ITC, VRC, MICO, FLC, and KTC), while A. fumigatus mdr4 has been shown to be active toward ITC (25). Large gene families allow members to diverge rapidly throughout evolution with the consequence of a loss or a gain of functions, resulting in a great diversity of substrates and specificities in the case of transporters. TruMDR1 and TruMDR3 are close to all identified *Candida* ABC azole transporters (34, 35).

**FIG 7:**
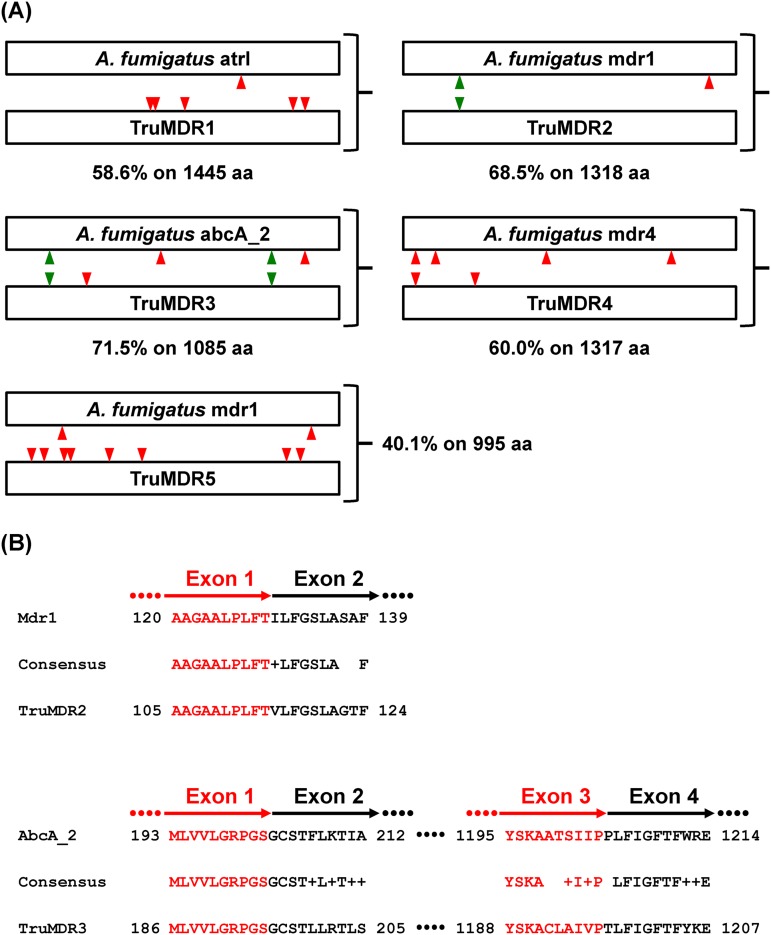
(A) Intron-exon structures of T. rubrum and A. fumigatus genes encoding ABC transporters. Green arrowheads indicate introns in ortholog positions. Red arrowheads indicate introns in other positions showing that the intron-exon structures of genes encoding T. rubrum azole ABC transporters and those of the closest homologs in A. fumigatus, are generally different. Refer to Table S3 for the amino acid positions in the transporter sequence, which correspond to the intron positions in the coding genes. (B) Comparison of the protein sequences encoded by the conserved intron region in *TruMDR2* and A. fumigatus *mdr1*, and comparison of the protein sequences encoded by the two conserved intron regions in *TruMDR1* and A. fumigatus *abcA*_2. The amino acid sequence coded by the 3′ extremity of the left exon is in red, and the amino acid sequence encoded by the 5′ extremity of the right exon is in black.

The intron-exon structures of genes encoding T. rubrum azole ABC transporters, and those of the closest homologs in A. fumigatus, were generally different (Fig. 7A and Table S3). The variability in the genetic models of ABC carriers cautions the use of the term orthologs between ABC transporter genes belonging to these fungal species. Only two pairs of genes could be considered orthologs, as follows: (i) *TruMDR3* and the closest A. fumigatus homolog *AFUA_2G15130*/*abcA_2*, which had three and four introns, respectively, in which the positions of introns 1 and 3 were conserved (Fig. 7B); and (ii) *TruMDR2* and A. fumigatus *mdr1*, which had one and two introns, respectively, in which intron 1 is conserved in the two genes (Fig. 7B). In contrast, TruMDR1 and the closest ABC transporter in A. fumigatus atrI (58% identity) cannot be considered orthologs. The unique intron in *TruMDR1* and the five introns contained in A. fumigatus *atrI* are in different positions (Fig. 7A). Likewise, *TruMDR4* (two introns) and *TruMDR5* (eight introns) cannot be considered the orthologs of A. fumigatus *mdr4* (four introns) and A. fumigatus *mdr1* (one intron), respectively (Fig. 7A and Table S3).

### TruMDR3 plays a major role in azole resistance of strain TIMM20092.

The disruption of *TruMDR3* in TIMM20092 abolished its resistance to VRC and reduced its resistance to ITC (Fig. 6). The reduced resistance to ITC is explained by the fact that the remaining TruMDR2, which is also significantly overexpressed in TIMM20092, is also an efflux pump for ITC, like TruMDR3 (Fig. 2). TruMDR3 was the only transporter to be inhibited by the multidrug resistance (MDR) inhibitor milbemycin oxime, and TIMM20092 lost its resistance to azoles after milbemycin oxime treatment. The sensitivity of TIMM20092 in the presence of milbemycin was comparable to that of the TIMM20092 ΔTruMDR3-33 and ΔTruMDR3-45 mutants (Fig. 6).

qRT-PCR analyses also showed that the gene encoding TruMDR3 was the most overexpressed in the azole-resistant strain TIMM20092, compared to the sensitive strain, CHUV1845 (Fig. 4A). Transcription of *TruMDR3* was upregulated upon exposure to ITC and VRC in TIMM20092 and in CHUV1845 (Fig. 4B and C). Altogether, these results were consistent and suggested that overexpression of *TruMDR3* was one of the major causes of the azole resistance in the TIMM20092 strain.

It should be noted that milbemycin inhibits both C. albicans CDR1 and CDR2 (14–16), whose amino acid sequences have approximately 50% identity with TruMDR3. TruMDR2 seems also to contribute to the azole resistance of TIMM20092, whereas TruMDR1 and TruMFS2 seem to play a weaker role in azole resistance of this clinical isolate. In contrast to findings by Martins et al. (13), *TruMDR4* was not found to be overexpressed upon exposure to ITC. Its expression in S. cerevisiae did not result in S. cerevisiae resistance to ITC, and therefore, this transporter does not appear to contribute to azole resistance in T. rubrum.

The molecular mechanisms leading to the overexpression of azole transporters have been described in pathogenic fungi other than dermatophytes. For instance, point mutations in C. albicans TAC1, a transcription factor that regulates *CDR1* and *CDR2*, were shown to mediate antifungal resistance through the overexpression of both genes encoding ABC transporters (45). A single base substitution in the *hapE* gene encoding the A. fumigatus transcription factor complex subunit led to azole resistance (46). Another A. fumigatus transcription factor, atrR, has been shown to play a pivotal role in a novel azole resistance mechanism by coregulating the drug target, Cyp51A, and putative drug efflux pump, abcC/cdr1B (47). The overexpression of genes coding for TruMDR2 and TruMDR3 leads to the assumption that there was a mutation gain of function in a transcription factor that remains to be identified in dermatophytes.

### Conclusion and perspectives.

Our study highlights the role of the efflux pumps, specifically, the ability of the ABC transporter TruMDR3 to counteract the effect of azole treatments, in the case of azole-resistant dermatophytosis. Additional transporters, including TruMDR2, might also contribute to the natural azole resistance of strain TIMM20092. *TruMDR2* and *TruMDR3* are both overexpressed in TIMM20092, and both transport ITC. Further disruption studies including the double disruption of *TruMDR2* and *TruMDR3* would provide more clues about the contribution of each transporter to ITC resistance. However, targeted gene disruption is not straightforward in T. rubrum and challenges the creation of such mutants.

Unlike the other transporters tested in this study, TruMDR3 was specifically inhibited by milbemycin oxime. In contrast, previous studies showed that milbemycin A3 and A4, as well as their oxime derivatives, repressed drug efflux by inhibiting more than one transporter in C. glabrata and C. albicans (16, 48, 49). Azole resistance could also be reduced with milbemycin oxime in Indian Trichophyton mentagrophytes strains recently sent for examination (data not shown), suggesting overexpression of the *MDR3* ortholog in these fungi. Dermatophyte isolates resistant to azole compounds or terbinafine have a high prevalence in India (9). The present study could be used as a basis for studying azole resistance in these isolates.

Milbemycin A3 and A4, as well as their oxime derivatives, are widely used in veterinary practice as antiparasitic drugs (50). A milbemycin derivative (moxidectin) was also used in humans to treat onchocerciasis (river blindness), a parasitic disease caused by the helminth Onchocerca volvulus (51). This study showed that such substances have low toxicity in humans. The drug resistance of fungal pathogens, including A. fumigatus, *Candida* species, and dermatophytes, poses a serious concern, given the limited number of antifungal drugs currently available (52–55). The increasing number of resistant isolates of pathogenic fungi in the world requires the development of new strategies to overcome resistance. Therefore, the combination of azoles with specific transporter inhibitors offers perspectives and potential solutions to treat dermatophyte infections in cases of azole resistance.

## MATERIALS AND METHODS

### Strains and plasmids.

T. rubrum TIMM20092 (6) and CHUV1845 were used in this study. The strains were stored as frozen stocks with 15% (vol/vol) glycerol at –80°C. S. cerevisiae strain Y02409 (*MAT***a** *ura*3Δ0 *leu2*Δ*0 his*3Δ1 *met15*Δ*0 YOR153w*::*kanMX4*; Euroscarf) and the expression vector pYES2 (Invitrogen-Life Technologies, Carlsbad, CA, USA) were used for heterologous expression of T. rubrum transporters. All plasmid subcloning experiments were performed in E. coli DH5α using the plasmid pUC57 (GeneCust, Ellange, Luxembourg). For *TruMDR3* gene disruption in T. rubrum TIMM20092, Agrobacterium tumefaciens EHA105 was maintained as previously described (56).

### Chemicals.

Itraconazole, milbemycin oxime, ibuprofen, farnesol, haloperidol, promethazine hydrochloride, and curcumin were purchased from Sigma-Aldrich (Buchs, Switzerland). Voriconazole (Vfend IV 200 mg) was acquired from Pfizer (Zurich, Switzerland). Milbemycin oxime and ibuprofen were dissolved in ethanol and distilled water (dH_2_O), respectively, at a concentration of 5 mg/ml. The other compounds were dissolved in dimethyl sulfoxide (DMSO; Sigma-Aldrich) to constitute stock solutions (5 mg/ml or 0.5 mg/ml for less soluble compounds). Stock solutions were stored at –20°C until use. Etest strips containing itraconazole (reference [Ref.] Etest, 0.002 to 32 μg/ml) and voriconazole (Ref. Etest, 0.002 to 32 μg/ml) were acquired from bioMérieux SA (Geneva, Switzerland) and stored at 4°C until use. Nonimpregnated cellulose disks (bioMérieux) were used for the diffusion assays.

### Growth media.

T. rubrum strains were grown on Sabouraud dextrose agar (SDA) and liquid medium (SDB) (Bio-Rad, Hercules, CA, USA). Liquid cultures were performed without shaking for 7 days at 30°C. Complete medium for S. cerevisiae consisted of 1% (wt/vol) Bacto yeast extract (Difco Laboratories, Detroit, MI, USA), 2% (wt/vol) Bacto peptone (Difco Laboratories), and 2% (wt/vol) dextrose (YPD). S. cerevisiae synthetic minimal medium (MMD) supplemented with histidine, leucine, methionine, and tryptophan (20 μg/ml) was prepared according to Sherman (57), with 2% (wt/vol) dextrose as the carbon source. Minimal medium with galactose (MMG) was prepared as MMD, but 2% (wt/vol) galactose was added as the carbon source instead of dextrose. MMG was used for the expression of genes cloned in the pYES2 expression vector under the control of the *GAL1* promoter. MMD and MMG plates were made with 2% (wt/vol) agar.

### Drug susceptibility assays for T. rubrum.

T. rubrum cultures were grown on 1/10 SDA (0.1% [wt/vol] peptone, 0.2% [wt/vol] dextrose, 2% [wt/vol] agar) for 14 days at 30°C. Sporulation was monitored by microscopy. T. rubrum spores were collected using sterile swabs and suspended in 3 ml of sterile dH_2_O. To obtain standardized conidial stock suspensions, absorbance values at a wavelength of 600 nm were determined (GeneQuant 1300 spectrophotometer; GE Healthcare Life Sciences, Marlborough, MA, USA) and diluted to a value of 1.0. The viable spore count was determined by inoculating serial decimal dilutions of the conidial stock suspensions on SDA plates. When considering the stock suspensions, an optical density (OD) value of 1.0 was found to correspond to 2.2 × 10^7^ to 2.3 × 10^7^ CFU/ml.

MICs were determined according to guidelines for the broth the microdilution method of the Clinical and Laboratory Standards Institute (CLSI) (38) but using SDB instead of RPMI medium (40). After incubation, the plates were read using a microtitration plate spectrophotometer (Multiskan Ascent OD595 nm; Thermo Fisher, Switzerland) set at a wavelength of 595 nm. The MIC_90_ was defined as the lowest concentration of the drug present in the wells showing growth inhibition of 90% or higher, in comparison to absorbance values obtained without antifungal drugs. For Etest assays, strips laced with an azole derivative were placed on fungal lawns (10^6^ CFU) prepared on SDA plates. For susceptibility assays on agar plates, 10 μl of serial dilutions of the stock suspensions (10^0^ to 10^3^, which corresponded to 10^5^ to 10^2^ spores, respectively) was spotted on SDA plates containing the desired concentration of antifungal drugs. The dishes were incubated at 30°C for 7 days.

### T. rubrum *cyp51A* sequencing.

T. rubrum genomic DNA was isolated from freshly growing mycelia using a DNeasy plant minikit (Qiagen, Hilden, Germany). A DNA fragment encoding T. rubrum Cyp51A was amplified by PCR with a standard protocol using homologous sense and antisense primers P1 and P2, respectively, (Table S4) and 200 ng of T. rubrum genomic DNA. DNA sequencing was performed by Microsynth (Balgach, Switzerland).

### T. rubrum cDNA library for heterologous expression in S. cerevisiae.

A cDNA bank of T. rubrum had already been prepared in the plasmid pSPORT6 (58). An RNA sample was prepared from T. rubrum, which was grown for 10 days in liquid soy protein medium (2 g soy protein [Supro 1711; Protein Technologies International] per liter), to favor the expression of genes encoding proteases (58–60). The cDNA fragments, which were cloned in pSPORT6, were then transferred to the pYES2-DEST52 plasmid using the Invitrogen Life Technologies Gateway technology for gene expression in S. cerevisiae.

### Bioinformatic identification of T. rubrum putative multidrug efflux pumps.

We performed a BLASTp analysis with A. fumigatus, C. albicans, and C. glabrata on T. rubrum recorded ABC and MFS transporters, which were known to confer azole and terbinafine resistance (Tables 1 and 2). Transporters in T. rubrum, with over 35% and 25% identity with any tested transporters of A. fumigatus and the *Candida* species, respectively, were retained for further investigations.

### Expression of T. rubrum ABC and MFS transporters in S. cerevisiae.

cDNA fragments encoding the MFS transporters TERG_04280, TERG_06399, TERG_07418, and TERG_08336 and all selected ABC transporters except for TERG_08612 were obtained by gene synthesis (GeneCust) and cloned into pUC57 between the BamHI and NotI sites of the multiple-cloning site. cDNA fragments encoding TERG_04626, TERG_05582, and TERG_07884 transporters were obtained by PCR using a standard protocol (56, 57), with the pairs of homologous primers P3-P4, P5-P6, and P7-P8, respectively (Table 1), and 200 ng of DNA prepared from 10^6^ clones of the cDNA library as a target.

Expression plasmids were constructed by cloning cDNA fragments encoding transporters into the vector pYES2 for gene expression in S. cerevisiae. Each pUC57 construct hosting a cDNA fragment was digested by the restriction enzymes BamHI and NotI, and the cDNA fragment was inserted end to end into the expression vector of S. cerevisiae, pYES2, and digested by the same enzymes. Likewise, PCR fragments for which BamHI and NotI sites were previously designed at the 5′ end of the primers were inserted into pYES2, digested by the same enzymes.

A DNA fragment encoding the TERG_08613 ORF was previously constructed as follows: the P9-P10, P11-P12, and P13-P14 sense and antisense primer pairs (Table S4) were used to amplify three contiguous fragments of T. rubrum genomic DNA. Subsequently, the three PCR products were digested with BamHI/NgoMVI, NgoMVI/XhoI, and XhoI/NotI, respectively, and inserted end to end into pYES2 digested with BamHI and NotI.

S. cerevisiae transformations were performed with 1.0 μg of pYES2 constructs, using a transformation kit (Invitrogen-Life Technologies) according to the manufacturer’s recommendations. The selection of *URA3* transformants was performed using MMD plates without uracil. Transformants were seeded onto MMG plates without uracil for heterologous expression of T. rubrum transporters under the control of the *GAL1* promoter.

### Drug susceptibility assays for S. cerevisiae transformants.

S. cerevisiae transformants were tested for antifungal resistance using serial dilution drug susceptibility assays and Etests. Yeasts were grown to mid-log phase (optical density at 600 nm [OD_600_], 1.0) at 30°C in liquid MMG, which contained the required amino acids. Each culture was diluted to an OD_600_ of 1.0. An OD value of 1.0 was found to correspond to about 10^7^ CFU/ml in the yeast suspensions. Subsequently, for the serial dilution drug susceptibility assays, 10 μl of serial dilutions (10° to 10^4^) was spotted onto MMG plates containing the desired concentration of antifungal drug.

For testing inhibitors on transporters, 10^6^ S. cerevisiae transformant cells were seeded to make a lawn on MMG agar-solidified medium plates, which incorporated a sub-MIC of ITC (0.015 and 0.0075 μg/ml) or VRC (0.02 μg/ml). Nonimpregnated cellulose disks, 6 mm in diameter, were then placed at the surface of the medium and individually saturated with 5 μg of each tested inhibitor. The plates were incubated at 30°C, and growth inhibition was observed after 5 days.

### Total RNA extraction and quantitative real-time reverse transcription-PCR.

T. rubrum CHUV1845 and TIMM20092 were grown in 50 ml of SDB, in 500-ml tissue culture flasks, without an antifungal drug and in the presence of 0.05 μg/ml of ITC or 0.02 μg/ml of VRC. Plugs from fresh fungal cultures were used as inoculates. Liquid cultures were conducted at 30°C, without shaking. After 7 days, the growing mycelia from each strain were collected, frozen, and ground under liquid nitrogen. Total RNA was extracted using the RNeasy plant minikit (Qiagen) and treated with DNase I (Qiagen). First-strand cDNA was synthesized using a high-capacity RNA-to-cDNA kit (Applied Biosystems, Carlsbad, CA, USA). The qRT-PCR analysis was performed using Power SYBR green PCR master mix on a StepOne real-time PCR system (Applied Biosystems) under standard conditions, according to the manufacturer’s recommendations.

We used primers designed by Martins et al. (13) to amplify *TruACT* (TERG_06637), *TruGAPDH* (TERG_04402), *TruMDR1* (TERG_02508), *TruMDR2* (TERG_08613), and *TruMDR4* (TERG_07801). The primers used to amplify *TruMDR3* (TERG_02186), *TruMDR5* (TERG_04952), *TruMFS1* (TERG_01623), and *TruMFS2* (TERG_08336) were designed in this work and are listed in Table S4. Dissociation curves of the qPCR-amplified products were plotted to confirm the absence of nonspecific products or primer dimers. The expression levels of the genes encoding transporters were examined as relative fold changes compared to their levels in the CHUV1845 strain. Gene expression was normalized to the actin gene (*TruACT*) using two primers, P15 and P16 (Table S4), and the relative quantification of gene expression was calculated according to the 2^ΔΔ^*^CT^* method (where *C_T_* is the threshold cycle). The statistical significance of *SQLE* gene expression levels among strains was evaluated using Student's *t* test.

### Construction of a transformation vector for targeted-gene disruption.

A *TruMDR3*-targeting vector, pAg1-*TruMDR3*/T (Fig. 5A and Table S5), was constructed as follows. Approximately 2.4 to 2.6 kb of the upstream and downstream fragments of *TruMDR3* (TERG_02186) was amplified from TIMM20092 total DNA by PCR with the P33-P34 and P35-36 primer pairs (Table S4), respectively. The *nptII* cassette, which is composed of the promoter sequence of Aspergillus nidulans *trpC* gene (*P_trpC_*), *nptII*, and the terminator of A. fumigatus *cgrA* gene (*T_cgrA_*), was amplified from the plasmid vector pSP72-PcFLP (Table S5) by PCR with the P44-45 primer pair (Table S4). The three amplified fragments were digested with SpeI/ApaI, BamHI/KpnI, or ApaI/BamHI and cloned into SpeI/KpnI doubly digested pAg1 (61) to generate pAg1-TruMDR3/T.

The PCRs were performed using PrimeSTAR high-sensitivity (HS) DNA polymerase (TaKaRa Bio). All of the internal ApaI, BamHI, KpnI, and SpeI sites contained in the amplified fragments were inactivated by overlap extension PCR with the P33-P37, P38-P39, P40-P34, P35-P41, and P42-P36 primer pairs (Table S4). If necessary, the amplified fragments were gel purified with a QIAEX II gel extraction kit (Qiagen), subcloned into HincII-digested pUC118, and sequenced.

### Fungal genetic transformation.

T. rubrum TIMM20092 was transformed by the A. tumefaciens-mediated transformation (ATMT) method, as described previously (56), with several minor modifications. After cocultivation, nylon membranes were transferred onto Sabouraud dextrose agar (SDA) medium containing 250 μg/ml G418 (Sigma-Aldrich) and overlaid with 10 ml of SDA supplemented with the same concentration of G418. The plates were further overlaid after 24 h with 10 ml of SDA containing 300 μg/ml G418 and then incubated for 6 to 7 days. The colonies regenerating on the selective medium were considered putative G418-resistant clones and transferred onto potato dextrose agar (PDA) medium supplemented with 100 μg/ml G418, 500 μg/ml cycloheximide, 50 μg/ml chloramphenicol, and 200 μg/ml cefotaxime sodium (Sanofi-Aventis), if necessary.

### Screening of the desired transformants.

The desired transformants were finally screened by PCR and Southern blotting. Total DNA was extracted according to a method described previously (56). Aliquots of 50 to 100 ng of the total DNA were used as the templates in the PCRs. For Southern blotting, aliquots of approximately 10 μg of the total DNA were digested with an appropriate restriction enzyme, separated by electrophoresis on 0.8% (wt/vol) agarose gels, and transferred onto Hybond-N^+^ membranes (GE Healthcare Ltd.). Southern hybridization was performed using an ECL Direct nucleic acid labeling and detection system (GE Healthcare Ltd.), according to the manufacturer’s instructions. Of the 5 T. rubrum transformants lacking the *TruMDR3* gene that were obtained, the ΔTruMDR3-33 and ΔTruMDR3-45 mutants were deposited as TIMM40001 and TIMM40002, respectively, in the culture collection of the Teikyo University Institute of Medical Mycology (TIMM).

### Data availability.

The updated sequences were submitted to GenBank with the following identification numbers: MK787243 (TERG_02186/*TruMDR3*), MK787253 (TERG_01443), MK787254 (TERG_02508/*TruMDR1*), MK787255 (TERG_04280), MK787256 (TERG_04952/*TruMDR5*), MK787257 (TERG_06399), MK787258 (TERG_07418), MK787259 (TERG_08336/*TruMFS2*), MK787260 (TERG_08591), MK787261 (TERG_08751), MK787262 (TERG_08613/*TruMDR2*), and MK787263 (TERG_07801/*TruMDR4*).

## Supplementary Material

Supplemental file 1
