# Supplementary material for: Trichophyton rubrum Azole Resistance Mediated by a New ABC Transporter, TruMDR3
Source: Antimicrob Agents Chemother. 2019 Oct 22;63(11):e00863-19. doi: 10.1128/AAC.00863-19 (PMC6811443; doi:10.1128/AAC.00863-19)
Supplement: Supplemental file 1 [file AAC.00863-19-s0001.pdf]

TABLE S1: Identification of potential *T. rubrum* (strain ATCC MYA-4607 / CBS 118892) genes coding for proteins containing an MFS transporter signature in UniProtKB

T. rubrum (strain ATCC MYA-4607 / CBS 118892)	**T. benhamiae* (strain ATCC MYA-4681 / CBS 112371)
TERG_02599/11867/868	ARB_02522
TERG_12004	ARB_04870
TERG_00675	ARB_05819
TERG_12306	ARB_05875
TERG_05381	ARB_03314
TERG_00820	ARB_07809
TERG_00215	ARB_05391
TERG_07783	ARB_06729
TERG_03719	ARB_00611
TERG_05391	ARB_03323
TERG_02614	ARB_02535
TERG_07492	ARB_00109
TERG_12412	ARB_04257
TERG_08394	ARB_00505
TERG_12018	ARB_04782
TERG_06400	ARB_04315
TERG_12178/179	ARB_01295
TERG_04094	ARB_07106
TERG_05575	ARB_05866
TERG_06880	ARB_00250
TERG_04400	ARB_00828
TERG_04625	ARB_02660
TERG_08244	ARB_01482

TERG_11942	ARB_05236
TERG_05532	ARB_05909
TERG_05810	ARB_06185
TERG_00348	ARB_05519
TERG_03813	ARB_00700
TERG_11583/584	ARB_05675
TERG_04308	ARB_00746
TERG_07230	ARB_02318
TERG_07539	ARB_00157
TERG_01336	ARB_01789
TERG_03066	ARB_05241
TERG_01255	ARB_07613
TERG_03216	ARB_05112
TERG_05526	ARB_05915
TERG_04949	ARB_03854
TERG_01456	ARB_02717
TERG_05582	ARB_05855
TERG_04626	ARB_02659
TERG_02583	ARB_02508
TERG_05429	ARB_05999
TERG_05055	ARB_03016
TERG_07040	ARB_01843
TERG_01532	ARB_03915
TERG_03055	ARB_05252
TERG_02689	ARB_03524
TERG_01437	ARB_01703
TERG_00574	ARB_05724
TERG_00162	ARB_02036

TERG_01820	ARB_06345
TERG_00916	ARB_01218
TERG_08400	ARB_00499
TERG_05125	ARB_03082
TERG_05537	ARB_05905
TERG_02992	ARB_05305
TERG_00008	ARB_02967
TERG_12369/370	ARB_04593
TERG_02822	ARB_07968
TERG_01053	ARB_03475
TERG_11599/600	ARB_05759
TERG_06717	ARB_02904
TERG_06093	ARB_04607
TERG_05351	ARB_03288
TERG_08336	ARB_02788
TERG_02783	ARB_07927
TERG_05547	ARB_05894
TERG_07884	ARB_06631
TERG_05199	ARB_03142
TERG_05891	ARB_06266
TERG_04711	ARB_03753
TERG_03240	ARB_05092
TERG_00776	ARB_07765
TERG_02295	ARB_07420
TERG_05104	ARB_03065
TERG_05466	ARB_05967
TERG_07298	ARB_00398
TERG_02844	ARB_07988

TERG_04399	ARB_00827
TERG_05309	ARB_03247/248
TERG_02323	ARB_07396
TERG_02369	ARB_07356
TERG_07170	ARB_02263
TERG_00163	ARB_02037
TERG_01634	ARB_01016
TERG_03011 ?	ARB_05289
TERG_05632	ARB_03543
TERG_07015	ARB_01819
TERG_07418	ARB_00048
TERG_04875	ARB_01436
TERG_01628	ARB_01022
TERG_03984	ARB_04003
TERG_05601	ARB_05841
TERG_02543	ARB_02464
TERG_00706	ARB_07695
TERG_01489	ARB_01528
TERG_02505	ARB_07219
TERG_02961	ARB_05332
TERG_01581	ARB_01061
TERG_05895	ARB_06270
TERG_04182	ARB_07021
TERG_04280	ARB_06932
TERG_01138	ARB_07500
TERG_01748	ARB_00908
TERG_03250	ARB_05083
TERG_08615	ARB_06758

TERG_06515	ARB_04208
TERG_02111	ARB_06624
TERG_07250	ARB_02337
TERG_06838	ARB_00288
TERG_03438	ARB_04908
TERG_12725/726	ARB_07858
TERG_08619	ARB_07866
TERG_05153	ARB_03107
TERG_06837	ARB_00289
TERG_08059	ARB_02098
TERG_05342	ARB_03279
TERG_01623	ARB_01027
TERG_03965	ARB_03984/985
TERG_01655	ARB_00993
TERG_08820	ARB_02699
TERG_04931	ARB_03838
TERG_01481	ARB_01519
TERG_04555	ARB_06847
TERG_03012	ARB_05288
TERG_02616	ARB_02537
TERG_02912	ARB_08054
TERG_02244	ARB_02435
TERG_02545	ARB_02467
TERG_05719	ARB_06094
TERG_05258	ARB_03197
TERG_08620	ARB_07865
TERG_04764	ARB_01340
TERG_06549	ARB_04173

TERG_06650	ARB_04081
TERG_06850	ARB_00278
TERG_02333	ARB_07387
TERG_02283	ARB_07432
TERG_01928	ARB_06441
TERG_02265	ARB_02449
TERG_01503	ARB_01540
TERG_08700	ARB_01015
TERG_06679	ARB_07177
TERG_03222	ARB_05108
TERG_03754	ARB_00644/645
TERG_03174	ARB_05150
TERG_05805	ARB_06180
TERG_12235/236	ARB_03026
TERG_04805	ARB_01378
TERG_12504	ARB_01831
TERG_08095	ARB_02130
TERG_04048	ARB_04075
TERG_03579	ARB_04786
TERG_02798	ARB_07943
TERG_05144	ARB_03099
TERG_05202	ARB_03145
TERG_04419	ARB_00852
TERG_01484	ARB_01522
TERG_11943	ARB_05235
TERG_12418	-
TERG_05549	-
TERG_06076	-

TERG_02654	-
TERG_05248	-
TERG_03451	-
TERG_04226	-
TERG_12503	-
TERG_07654	-
TERG_01912	-

* Closest homologs in *Trichophyton benhamiae* (formerly *Arthroderma benhamiae*) strain ATCC MYA-4681/CBS 112371 are also indicated when found.

TABLE S2: Identification of potential *T. rubrum* (strain ATCC MYA-4607 / CBS118892) genes coding for proteins containing an ABC transporter signature in UniProtKB

T. rubrum (strain ATCC MYA-4607 / CBS 118892)	**T. benhamiae* (strain ATCC MYA-4681 / CBS 112371)	ABC Family
TERG_12478	ARB_00234	ABCC
TERG_12475	ARB_00239	ABCG
TERG_03793	ARB_00681	**ABCF
TERG_04309	ARB_00747	ABCB
TERG_04323	ARB_00761	ABCC
TERG_00955	ARB_01184	ABCG
TERG_04806	ARB_01379	ABCG
TERG_04848	ARB_01416	ABCC
TERG_08693	ARB_01506/507	ABCB
TERG_01443	ARB_01699	ABCC
TERG_01420	ARB_01716	n.d
TERG_12650/651	ARB_02150	ABCC
TERG_08130	ARB_02160	**ABCF
TERG_02186	ARB_02379	ABCG
TERG_04628	ARB_02657	**ABCF
TERG_08299	ARB_02755	ABCC
TERG_05126	ARB_03083	ABCB
TERG_05254	ARB_03193	**ABCE
TERG_03878	ARB_03588	ABCD
TERG_04952	ARB_03857	ABCB
TERG_03933	ARB_03963	ABCC
TERG_03988	ARB_04007	**ABCF
TERG_06399	ARB_04316	ABCB

TERG_06212	ARB_04497	ABCB
TERG_12373	ARB_04544	ABCC
TERG_00286	ARB_05462	ABCB
TERG_00402	ARB_05580	ABCB
TERG_05538	ARB_05904	ABCB
TERG_05776	ARB_06148	ABCD
TERG_07801 ?	ARB_06709	ABCB
TERG_08613	ARB_06760	ABCB
TERG_08591	ARB_06781	ABCB
TERG_02508	ARB_07216	ABCG
TERG_01131	ARB_07493	ABCB
TERG_01274	ARB_07630	**ABCF
TERG_11627	ARB_07762	ABCB
TERG_08751	ARB_07873	ABCB
TERG_04224	-	ABCA
TERG_04227	-	ABCG

*Closest homologs in *T. benhamiae* (formerly *Arthroderma benhamiae*) strain ATCC MYA-4681 / CBS 112371 are also indicated when found.

**Families ABCE and ABCF do not have the transmembrane domains and are probably not transmembrane transporters.

TABLE S3: Gene model of *TruMDR1-5* and of closest *A. fumigatus* homologs.

***T. rubrum* ABC trasporter**	**Size in aa**	**Poition of introns***	**Closest homologue of *A. fumigatus***	**Size in aa**	**Amino acid positions corresponding to introns***	**Sequence identities ****
TERG_02508 / *TruMDR1*	1270	596<br>597<br>708<br>1236<br>1566	AFUA_3G07300 / atrI	1472	998	58.6% on 1445 aa
TERG_08613 / *TruMDR2*	1331	114	AFUA_5G06070 / mdr1 / abcA	1349	129<br>1231	68.5% on 1318 aa
TERG_02186 / *TruMDR3*	1503	195<br>373<br>1197	AFUA_2G15130 / abcA_2	1449	202<br>706<br>1204<br>1359	71.5% on 1085 aa
TERG_07801 / *TruMDR4*	1366	17<br>337	AFUA_1G12690 / mdr4	1330	23<br>62<br>529<br>1083	60.0% on 1317 aa
TERG_04952 / *TruMDR5*	1292	63<br>90<br>126<br>139<br>345<br>488<br>964<br>1008	AFUA_5G06070 / mdr1 / abcA	1349	129<br>1231	40.7% on 995 aa

* Amino acid positions in the sequence of transporters corresponding to the positions of introns in coding genes. The amino acid positions referring to conserved introns are marked in bold.

** Protein sequence identities are indicated with the length of the identical parts of the proteins.

TABLE S4: Primers used in this study

*Primers	Oligonucleotide sequence	Location	PCR product size with cloning sites<br>Purpose
P1<br>P2	5′-ATGGCCGTGCTCACAGTGG-3′<br>5′-TCAACGTGAATTAGAACGTCG-3′	TERG_02984<br>complement of TERG_02984	1615 bp
P3<br>P4	5′-GTT*GGATCC*AACATGGTGTCTGACATCGAGAAG-3′<br>5′-CTT*GCGGCCGC*TACATATCTCGCTCCATTTCC-3′	TERG_04626<br>Complement of TERG_04626	1835 bp<br>*Bam*HI---*Not*I<br>Cloning in pYES2
P5<br>P6	5′-GTT*AGATCT*CAACATGGCCACTTTTCTTCCCAGC-3′<br>5′-CTT*GCGGCCGC*CTACTCCGCAAATCGACTTCTT-3′	TERG_05582<br>Complement of TERG_05582	1734 bp<br>*Bgl*II---*Not*I<br>Cloning in pYES2
P7<br>P8	5′-GTT*AGATCT*CAACATGGCGAATTCATCCGGCTCGG-3′<br>5′-CTT*GCGGCCGC*CTATTTCTTTTCAGAGTTTTCC-3′	TERG_07884<br>Complement of TERG_07884	1638 bp<br>*Bgl*II---*Not*I<br>Cloning in pYES2
P9<br>P10	5′-GTTT*GGATCC*AACATGGTTGAACCGAGCGAGAAAC-3′<br>5′-ATGT*GCCGGC*GAGGGAACCAAAAAGGACTGTAAATAGC GGTAGGGCGGC-3′	TERG_08613<br>Complement of TERG_08613	383 bp<br>*Bam*HI---*Ngo*MIV<br>Cloning in pYES2
P11<br>P12	5′-GGTTCCCTC*GCCGGC*ACATTTC-3′<br>5′-CTTT*CTCGAG*CACCGCCTCATCCTCGG-3′	TERG_08613<br>Complement of TERG_08613	1708 bp<br>*Ngo*MIV ---*Xho*I<br>Cloning in pYES2
P13<br>P14	5′-GGATGAGGCGGTG*CTCGAG*AAGGAGAAGG-3′<br>5′-CTAT*GCGGCCGC*TTAATGAGTTTTCTCAAGGCTC-3′	TERG_08613<br>Complement of TERG_07884	1972 bp<br>*Xho*I---*Not*I<br>Cloning in pYES2
P15<br>P16	5′-TGTTGGTGATGAGGCACAGT-3′<br>5′-CCATGTCATCCCAGTTTGTG-3′	TERG_06637: *TruACT* (actin)<br>Complement of TERG_06637	89 bp<br>qRT-PCR

P17<br><br>P18	5′-GCGTGACCCAGCCAACA-3′<br><br>5′-TTGGCACCTCCCTTCAAGT-3′	TERG_04402: *TruGAPDH* Complement of TERG_04402	114 bp<br><br>qRT-PCR
P19<br><br>P20	5′-CCTAATGCCCTTCCTGGATT-3′<br><br>5′-AAATTGCCAGCTCGTTCTGT-3′	TERG_02508: *TruMDR1* Complement of TERG_02508	130 bp<br><br>qRT-PCR
P21<br><br>P22	5′-TGACTCTGAATCCGAAAAGG-3′<br><br>5′-GTCGGTGAGCAACAGCAATA-3′	TERG_08613: *TruMDR2* Complement of TERG_08613	83 bp<br><br>qRT-PCR
P23<br><br>P24	5′-TCACTGGTGAAATGCTCGTTGACG-3′<br><br>5′-GGTTGACGGAGCAAGGCACTG-3′	TERG_02186: *TruMDR3* Complement of TERG_02186	142 bp<br><br>qRT-PCR
P25<br><br>P26	5′-GGAAATTGAGCTTCGAGACG-3′<br><br>5′-TTCCAACGATAGCAGTGTGC-3′	TERG_07801: *TruMDR4* Complement of TERG_07801	113 bp<br><br>qRT-PCR
P27<br><br>P28	5′-ATCGGGCACTGCTTTGCCTC-3′<br><br>5′-GCAGATGGTATGGATGTAGACG-3′	TERG_04952: *TruMDR5* Complement of TERG_04952	181 bp<br><br>qRT-PCR
P29<br><br>P30	5′-ATTATCGGCCGTGCAGTTGC-3′<br><br>5′-ACCTGCCACAGAGGCAATACC-3′	TERG_01623: *TruMFS1* Complement of TERG_01623	147 bp<br><br>qRT-PCR
P31	5′-GCTGATGCCAATGCCATGGAG-3′	TERG_08336: *TruMFS2*	178 bp

P32	5′-TCGGGTTTCGAGGATCGCTTGG-3′	Complement of TERG_08336	qRT-PCR
P33<br>P34	5′-TGT*ACTAGT*ACCGTGGCCATCGTGTAGAGGATATT-3′<br>5′-TCGG*GGGCCC*GCTGCTGATCTCTATGGACGATAGC-3′		2627 bp<br>Amplification of *TruMDR3*-5'-UTR
P35<br>P36	5′-AAAG*GGATCC*TTCTATTTCAATTGGAATACTCGAT-3′<br>5′-CGAT*GGTACC*AAAACTCGAGACTCAACTCTCACCC-3′		2407 bp<br>Amplification of *TruMDR3*-3'-UTR<br>Amplification of a hybridization probe (P35)
P37<br>P38<br>P39<br>P40<br>P41<br>P42	5′-GACCGAGGCATCAGGGATCTGAGCAGGCAGGTTGG-3′<br>5′-CCAACCTGCCTGCTCAGATCCCTGATGCCTCGGTC-3′<br>5′-CAGCGTATGTTCAGGGTCCAATCATATGCCATCGA-3′<br>5′-TCGATGGCATATGATTGGACCCTGAACATACGCTG-3′<br>5′-TGTCAACAAACTAACTGGTTAACCTTTCGTCCGAT-3′<br>5′-ATCGGACGAAAGGTTAACCAGTTAGTTTGTTGACA-3′		Inactivation of the internal *Apa*I, *Bam*HI, *Kpn*I or *Spe*I sites within the 5'- or 3'-UTR by overlap extension PCR
P43	5′-TCGTTCATGGACAATGTGAAAGCCACCCATGTGTT-3′		509 bp<br>Amplification of a hybridization probe
P44<br>P45	5′-TTTT*GGGCCC*AGAAGATGATATTGAAGGAGCACT-3′<br>5′-AAAA*GGATCC*AGATGATTCATGA-3′		1451 bp<br>Amplification of *nptII* cassette (PtrpC-*nptII*-TcgrA)

*Primers P1 and P2 were used for *T. rubrum cyp51A* (TERG_02984) amplification and sequencing. Primers P3 to P8 were used for amplification of genes to be expressed in *S. cerevisiae*. CAAC Kozak sequences are underlined. P9 to P14 were used for a *TruMDR2* (TERG_08613) cDNA fragment synthesis. P15 to P32 were used for qRT-PCR experiments. P33 to P45 were used for construction of *TruMDR3*-targeting vector pAg1-*TruMDR3*/T and *TruMDR3* locus-disruption experiments in TIMM20092.

TABLE S5: Plasmids used for constructing the *TruMDR3*-targeting vector pAg1-*TruMDR3*/T

**Plasmid**	**Description**	**Source**
pAg1	A streamlined version of the binary vector pBIN19 containing sequences necessary for replication in *E. coli* and *A. tumefaciens* (*oriV*, *trfA*), *E. coli* neomycin phosphotransferase (*nptII*), and the transferable DNA (T-DNA) region. A multiple cloning site was placed within the T-DNA region.	1
pSP72-PcFLP	pSP72 (Promega) containing the 5'-UTR of *Trichophyton mentagrophytes Ku80* (*TmKu80*) gene (AB427108) (1.5 kb), the FLP recombination target (5'-FRT), *Aspergillus nidulans trpC* promoter (*PtrpC*) (AN0648), *nptII*, *A. fumigatus cgrA* terminator (*TcgrA*) (AFUA_8G02750), *T. rubrum ctr4* promoter (*Pctr4*) (TERG_01401), *Penicillium chrysogenum*-optimized FLP recombinase (*Pcflp*) gene, *Cryptococcus neoformans trp1* terminator (*Ttrp1*) (M74901), 3'-FRT and the 3'-UTR of *TmKu80* gene (1.5 kb).	This study
pAg1-*TruMDR3*/T	*TruMDR3a* fragment [the 5'-UTR of *TruMDR3 gene* (TERG_02186); 2.6 kb], *PtrpC*, *nptII*, *TcgrA*, *TruMDR3b* fragment (the 3'-UTR of *TruMDR3* gene; 2.4 kb)	This study

<u>Reference</u>

1. Zhang A, Lu P, Dahl-Roshak AM, Paress PS, Kennedy S, Tkacz JS, An Z. 2003. Efficient disruption of a polyketide synthase gene (pks1) required for melanin synthesis through *Agrobacterium*-mediated transformation of *Glarea lozoyensis*. Mol Genet Genomics 268:645-655.
